# Social Type-Aware Navigation Framework for Mobile Robots in Human-Shared Environments

**DOI:** 10.3390/s24154862

**Published:** 2024-07-26

**Authors:** Sumin Kang, Sungwoo Yang, Daewon Kwak, Yura Jargalbaatar, Donghan Kim

**Affiliations:** 1 AgeTech-Service Convergence Major, Department of Electronic Engineering, Kyung Hee University, Yongin 17104, Republic of Korea; suminsk@khu.ac.kr (S.K.); p1112007@khu.ac.kr (S.Y.); 2 Department of Artificial Intelligence, Kyung Hee University, Yongin 17104, Republic of Korea; kdw1181@khu.ac.kr

**Keywords:** autonomous mobile robot, human–robot interaction, proxemics, social robotics

## Abstract

As robots become increasingly common in human-populated environments, they must be perceived as social beings and behave socially. People try to preserve their own space during social interactions with others, and this space depends on a variety of factors, such as individual characteristics or their age. In real-world social spaces, there are many different types of people, and robots need to be more sensitive, especially when interacting with vulnerable subjects such as children. However, the current navigation methods do not consider these differences and apply the same avoidance strategies to everyone. Thus, we propose a new navigation framework that considers different social types and defines appropriate personal spaces for each, allowing robots to respect them. To this end, the robot needs to classify people in a real environment into social types and define the personal space for each type as a Gaussian asymmetric function to respect them. The proposed framework is validated through simulations and real-world experiments, demonstrating that the robot can improve the quality of interactions with people by providing each individual with an adaptive personal space. The proposed costmap layer is available on GitHub.

## 1. Introduction

Recent advances in robotics technology have enabled autonomous mobile robots to be used in various places. Many robots already perform in social spaces where they coexist with humans, such as museums, airports, and hotels [1,2]. When navigating social spaces, robots must avoid obstacles and humans safely. In this context, conventional mobile robot navigation systems consider humans as dynamic obstacles and focus on avoiding physical collisions [3,4,5]. However, humans still feel discomfort or lack trust in such robot behavior. Therefore, it is crucial for robots to avoid physical collisions with humans and ensure that humans feel safe and comfortable with the robot’s behavior [6]. Proxemics is a field of psychological research that studies how people use the physical space around them and how they position themselves concerning each other [7]. When people navigate an environment and engage in social interactions, they subconsciously consider the personal space of others. They may feel uncomfortable if the distance between two people is closer than expected. Robots should also respect these social norms in social spaces and consider people’s personal space [8]. In this context, many researchers are applying proxemics concepts to robot navigation, and this approach has been studied in Refs. [9,10,11].

Most studies treat all people the same and do not consider different age groups and situations. However, in the real world, children and adults have different social and physical characteristics, and people move in different patterns, such as moving alone or in groups. In particular, children have less ability to react to accidents than adults, which can lead to safety issues when interacting with robots [12,13]. Ref. [14] investigated human–robot interaction and found that children tend to keep more distance than adults when interacting with a robot. In this context, applying a one-size-fits-all avoidance strategy to everyone without considering diversity can make people uncomfortable with the robot’s behavior or lead to unexpected situations. Robots should adopt different avoidance strategies for different people to feel more comfortable and safer in their interactions. We propose a novel Social Type-Aware Navigation Framework for robots to directly detect and track people in real-world environments and adapt avoidance strategies for each individual.

**Figure 1:**
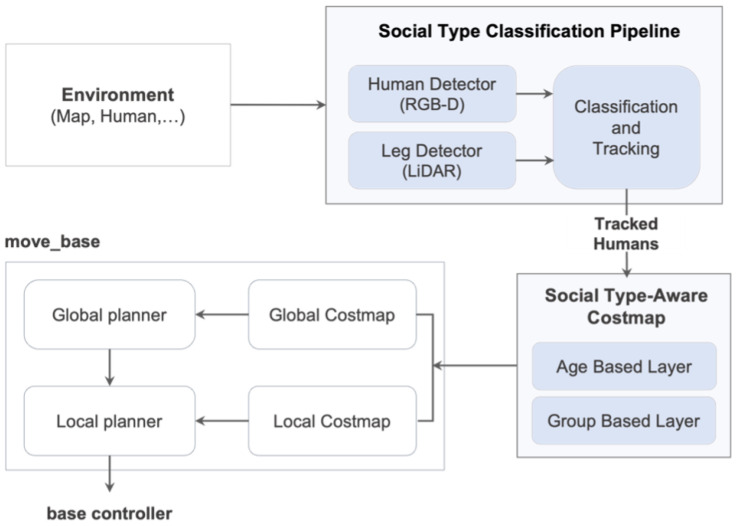
An overview of the Social Type-Aware Navigation Framework.

**Figure 2:**
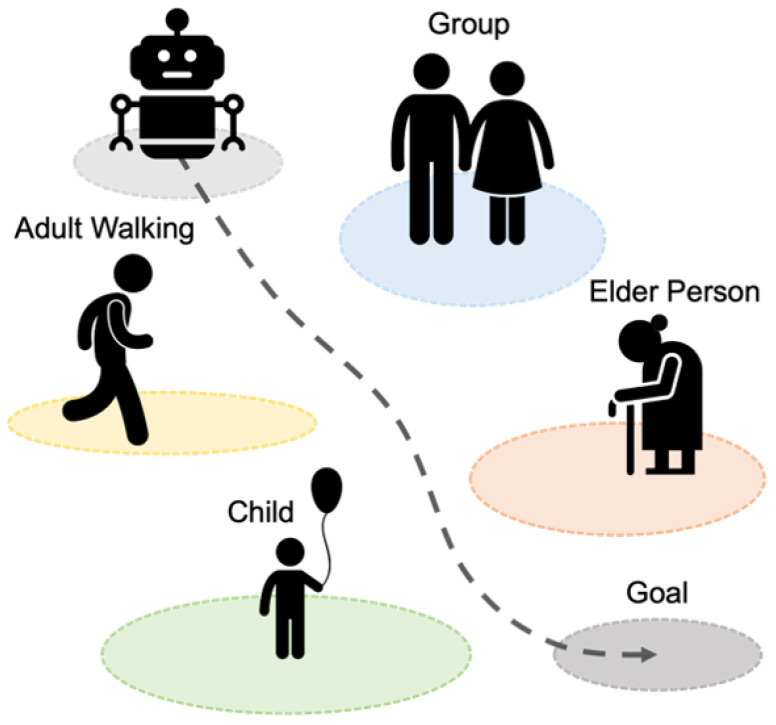
Social Type-Aware Navigation Framework for comfort in social spaces.

Thus, we propose a framework that enables robots to move safely in social spaces between different ages and types of people, including socially vulnerable people such as children and the elderly, while understanding their personal space and social context. To this end, the framework includes a ‘Social Type Classification Pipeline’ that enables robots to detect people in real-world environments directly, classify them into different social types, and a ‘Social Type-Aware Costmap (STAC)’ that enables them to navigate while respecting the appropriate social distance according to each classified social type. Our framework is illustrated in Figure 1 and is designed as a plugin that can be easily integrated into an ROS-based navigation system, allowing it to be used with various path planning methods. Applying our framework enables robots to move among people more naturally and in a socially contextualized manner, as shown in Figure 2. Our main contributions to this work can be summarized as follows:

1. We present a pipeline using robot sensors to classify humans into diverse social types, moving beyond the conventional approach of treating all humans the same.
2. We develop a new layered costmap that adjusts personal space for each social type, enabling robots to navigate more sensitively and safely around humans.
3. We validate our framework through qualitative and quantitative analysis in simulated and real-world human–robot interaction scenarios and demonstrate its performance.

This paper is organized as follows: Section 2 describes the background and necessity of this research by reviewing the related works. Next, Section 3 details the Social Type-Aware Navigation Framework proposed in this paper. In Section 4, we qualitatively and quantitatively analyze the results of the experiments conducted with human–robot interaction scenarios in simulated and real-world environments to validate the performance of the proposed framework. Finally, Section 5 summarizes the contributions of this work and concludes the paper with suggestions for future works.

## 2. Related Work

### 2.1. Human Detection and Tracking

In dynamic and unknown environments, detecting nearby obstacles and people through sensors is essential for robots to navigate safely to their goal. LiDAR and cameras are commonly used for mobile robots, and LiDAR is useful for detecting humans by tracking the geometric features of their legs [15]. However, there is a limitation in identifying human features, such as age group, using only LiDAR.

On the other hand, with the improvement of computer vision systems, algorithms such as R-CNN [16], Faster R-CNN [17], and YOLO [18] have made it possible to detect people in camera images. For example, Ref. [19] presented a method to estimate the age and gender of a human in an image using YOLO. This approach was used in Ref. [20] to recognize the gender of a customer so that a robot could interact with them in a shopping center. Ref. [21] proposed a face detection and tracking framework using convolutional neural networks and the SORT [22] for integration into various real-world HRI systems. However, these methods mainly rely on frontal faces, which are less accurate for the side faces or small-sized faces due to the field of view (FoV) of the camera sensor on the mobile robot. To address this challenge, Refs. [23,24] proposed a novel approach to distinguish between adults and children by calculating the ratio of the torso to the face.

In public spaces and crowded environments, robots need to recognize when people are in groups to plan safer and more efficient paths and to adjust their interactions with people properly. In this context, many researchers have proposed many different methods to detect groups in images. In Ref. [25], the sensor was placed overhead to detect group interactions between people, i.e., from an exterior perspective, but it did not represent the robot vision and was challenging to implement in real-world navigation. Therefore, Refs. [26,27] detected groups from the robot perspective in real time by object detection and clustering using cameras installed on the robot.

Mobile robots should be able to track humans in real time and predict their trajectories to reasonably plan their path so that they can complete tasks more efficiently. To achieve this, Ref. [28] proposed a multi-modal tracking framework that integrates various tracking methods such as Nearest-Neighbor [29], Extended Nearest-Neighbor [30], Multi-Hypothesis [31], and Vision-Based MDL [32] to improve human tracking in crowded environments. Also, in Ref. [33], they used a real-time tracking algorithm, SORT [22], based on a Kalman filter to identify the bounding boxes captured by a camera to track a human continuously. These studies contribute to predicting human movements when a mobile robot operates in a social space.

### 2.2. Proxemics in Human–Robot Interaction

When a robot needs to navigate around humans, it is important to consider proxemic rules to understand and respond appropriately during interactions with humans. For example, Ref. [34] evaluated subjects’ reactions to the robot’s avoidance behavior with real-world experiments in which a robot and a human encountered each other in a hallway. They found that the subjects felt more comfortable when the robot kept away from them. Also, Ref. [35] defined the shape of personal space by evaluating the comfort humans feel with the movement of the humanoid and non-humanoid robots when passing around the person. These results suggest that robots should respect their personal space and behave predictably to ensure comfort and safety while interacting with humans. Ref. [11] developed this concept in this context and modeled a personal space as an asymmetric Gaussian function. Refs. [36,37] integrated the Gaussian function from Ref. [11] into a costmap by adding a cost around a person to make the robot respect their personal space. The research in Ref. [38] developed a dynamic social force model, which determines the parameters of personal space with a fuzzy inference system and continuously adjusts them through reinforcement learning. In Refs. [39,40], the robot detected human emotions and incorporated emotion-based proxemics constraints into the costmap. This approach makes human–robot interactions smoother by allowing the robot to recognize human emotions and react appropriately.

Meanwhile, human interaction in crowded environments is not limited to interactions between individuals. For example, humans tend to avoid groups they are not involved with. In this context, it is crucial for robots to understand the interactions with human groups and react accordingly. That is, robots should recognize the spaces generated by groups of humans and navigate among them effectively. Kendon’s F-formation [41] is a good tool for understanding the interactions between such groups, including the o-space shared by group members, the p-space where group members stand, and the R-space that divides the group from outer space. Refs. [42,43,44,45,46] took this idea and proposed an approach in which the robot maintains an appropriate distance from the group so that the individual as well as the group members feel comfortable, and Refs. [47,48] addressed how the robot detects groups and approaches them with an appropriate pose when joining the group.

Although many studies have applied proxemics to social navigation, a lack of research defines and considers personal space for children. By categorizing people into different age groups or situations instead of a single category and respecting their different personal spaces, a robot can navigate safely and be human-aware in real-world environments. This differentiated approach is an important step forward. It will make robots interact with all kinds of humans, including children.

## 3. Methods

### 3.1. Social Type Classification Pipeline

Many studies in the area of socially aware navigation use ceiling-mounted cameras to detect people [49]. In this work, we developed a pipeline to detect, track, and classify people using sensors installed on a robot for applications in various real-world environments.

**Figure 3:**
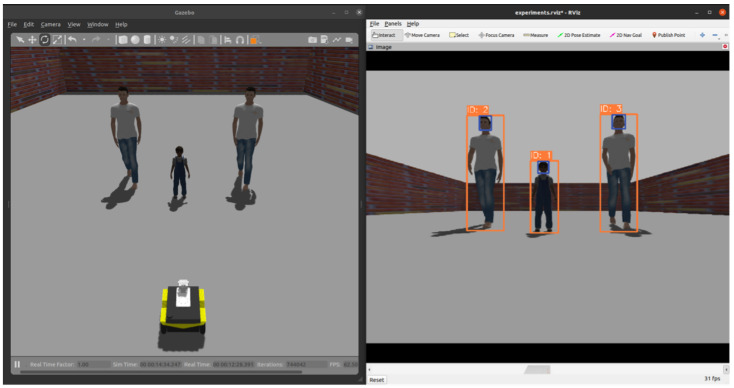
Human detection and tracking result in a Gazebo environment.

First, we chose Ultralytics’ open-source YOLOv7 model [50] to detect people in RGB images. It provides high accuracy and fast processing speed and improves performance compared to existing models. We trained YOLOv7 on the CrowdHuman dataset [51], which provides three annotated datasets: human visible area, head, and full body. Applying the trained model, we can see that the bounding boxes for the head and the whole body are detected, as shown in Figure 3.

For the tracking system, we use SORT (Simple Online and Realtime Tracking) [22]. This algorithm tracks each uniquely identified object through the scene and can re-identify them even if they leave and re-enter the scene. The output of this system includes an ID for each object, along with its start and end positions in terms of x and y coordinates. These coordinates can be used to analyze the movement and path of each person. By analyzing the history of these positions, the system can estimate a person’s speed and trajectory, providing valuable insight into their movement patterns within the scene.

Figure 3 shows the results of detecting and tracking people using YOLOv7 and SORT in a Gazebo environment. The left image shows the Gazebo and the right image shows the detection and tracking results. The blue bounding boxes indicate detections of heads and the orange ones full bodies. The top of each orange bounding box also shows the ID of the detected person, which can be used to effectively distinguish between multiple people in the image.

To estimate the age of the detected person, we use the human head-to-body ratio [52]. Since there is a difference in this ratio for each age group, we can identify adults and children by comparing the size of the head and body bounding boxes of each detected person. If multiple humans are detected, classification is performed by ensuring that the center of the head bounding box is within the full body bounding box before calculating the ratio of the bounding boxes. The threshold for distinguishing between adults and children based on their head-to-body ratio was determined through prior experiments in various real-world settings. A ratio of 7.0 or less was classified as a child, while a ratio greater than 7.0 was classified as an adult. This classification ensures that the robot moves carefully when interacting with children.

After detecting and classifying humans, we use the optical principle of RGB-D cameras to determine their actual location. From the depth image extracted from the RGB-D image, the bounding box and the internal parameters of the camera can be used to convert the position of the human from the 2D image to 3D coordinates in the camera coordinate system. This conversion is calculated as follows:

(1) $$\;X_{C}=\frac{u-p_{x}}{f_{x}}Z_{C}\;,\;Y_{C}=\frac{v-p_{y}}{f_{y}}Z_{C}\;,\;Z_{C}=Z$$

In Equation (1), (u,v) is the center coordinate of the 2D bounding box, $\left(p_{x},p_{y}\right)$ is the principal point coordinate of the camera, $\left(f_{x},f_{y}\right)$ are the focal lengths of the camera, *Z* is the value of the corresponding pixel in the depth image, and $\left(X_{C},Y_{C},Z_{C}\right)$ represents the 3D coordinate in the camera coordinate system. Finally, the actual human position can be obtained through a coordinate transformation between the camera and robot coordinate systems.

**Figure 4:**
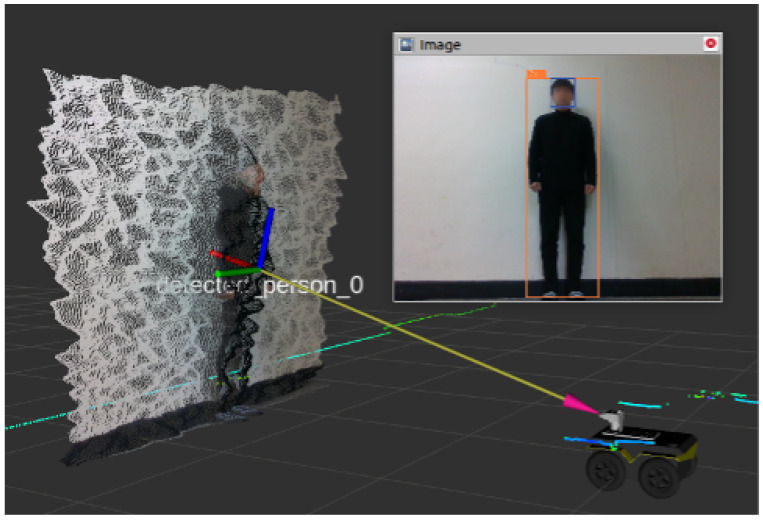
Example of the human position detected using the RGB-D camera in the robot coordinate system.

Figure 4 shows the result of the human position detected by the RGB-D camera in the robot coordinate system. It shows the process of detecting a person in the RGB image from the camera on the robot, combining the depth information of the corresponding bounding box with the internal parameters of the camera and converting the position in the 2D image to 3D spatial coordinates in the robot coordinate system. When a robot is navigating around people, the robot must know the actual position and movement of the person, which allows the robot to predict the future trajectories of the person and thus avoid collisions with the person in advance. In this paper, to estimate the human’s motion and obtain the human’s exact position, we use the Kalman filter [53].

The robot we use has a 90-degree field of view for the camera and a 270-degree field of view for the LiDAR. Since the field of view of the RGB-D sensor covers only 1/3 of the laser scanner’s field of view, we use Ref. [15], which estimates the position and speed of people with the LiDAR sensor, to recognize all the people around the robot.

There are not only individuals but also human groups in social space. Many researchers proposed a group identification method using clustering techniques in this context. However, these methods are limited in real-time applications due to data processing. Therefore, we propose a simple group detection algorithm that can recognize groups in a dynamic environment. According to proxemics, people tend to be distant from strangers more than familiar people. The algorithm calculates the differences in distance and direction of movement between people. It classifies them into a group if they move in the same direction and the distance is within a threshold range based on proxemics.

Algorithm 1 shows the proposed group recognition algorithm for recognizing groups based on individuals’ spatial and orientation data. It utilizes two pieces of information about each individual: their location, denoted by $\left(x_{i},y_{i}\right)$, and their orientation, represented by the quaternion $q_{i}$, to detect whether they are in a group.

The process starts by converting the quaternion into a rotation matrix (using the RotMat function) so that the orientation of each individual can be easily used for spatial analysis. This transformation is essential for determining each individual’s heading vector, which enables us to know where each individual is facing. Next, it calculates the distance and angular difference between individuals. The distance is determined using the Euclidean distance formula, and the angle is a simple calculation obtained by comparing the orientation vectors derived from the rotation matrix (using the VecAngle function). The final step is to apply a predefined threshold to these calculated distances and angles to classify objects as part of a group or as individual objects. If the distance and angle differences are within this threshold, they are considered to belong to the same group.
**Algorithm 1** Group Classification1:**Input:** For each human $p_{i}$, position as $p_{i}=\left(x_{i},y_{i}\right)$, orientation as quaternion $q_{i}$2:**Output:** Classification of each pair as a group or individuals3:**function** RotMat(q)4:  Compute rotation matrix from quaternion *q*5:  **return** rotation matrix6:**end function**7:**function** VecAngle(v1, v2)8:   u1←v1/∥v1∥9:   u2←v2/∥v2∥10:  dp← dot product of u1 and u211:  angle←arccos(max(−1,min(1,dp)))12:  **return** angle13:**end function**14:**for** i=1 to n−1**do**                ▹ For each $p_{i}$ in people15:  **for** j=i+1 to *n***do**           ▹ For each $p_{j}$ in people after $p_{i}$16:   $dist\leftarrow \sqrt{{\left(x_{j}-x_{i}\right)}^{2}+{\left(y_{j}-y_{i}\right)}^{2}}$17:   $r_{i}\leftarrow RotMat\left(q_{i}\right)$18:   $r_{i}\leftarrow RotMat\left(q_{j}\right)$19:   $d_{i}\leftarrow r_{i}\cdot \left[1,0,0\right]$                ▹ Direction vector of $p_{i}$20:   $d_{j}\leftarrow r_{j}\cdot \left[1,0,0\right]$                ▹ Direction vector of $p_{j}$21:   $angle\leftarrow VecAngle(d_{i},d_{j})$22:   **if** (dist<distThresh) and (angle<angleThresh) **then**23:    Classify $p_{i}$ and $p_{j}$ as a group24:  **end if**25: **end for**26:**end for**

In conclusion, our pipeline becomes an essential basis for the Social Type-Aware Costmap (STAC).

### 3.2. Social Type-Aware Costmap (STAC)

Costmap [54] is a data structure that represents the environments around the robot in the form of a 2D grid map, where each grid cell represents the ’cost’, or ’risk’, of the position. The cost represents the expected risk of the robot navigating through the area. The robot can choose a safe and efficient path by avoiding high-cost areas and preferring low-cost areas. Costmaps are widely used for socially aware motion planning and navigation by introducing non-lethal costs to represent social space [55]. Ref. [36] proposed a layered costmap, each containing semantic information about specific attributes such as obstacles, inflation, or proxemics. They represent proxemics by utilizing the detected position and velocity of humans to generate Gaussian distributions of costs around them [11].

While these approaches effectively represent human personal space, they must consider that it can vary from person to person. Hence, in this work, we propose a Social Type-Aware Costmap (STAC), a multi-layer costmap that includes the personal space for each type of person, such as adults, children, and groups. Our costmap allows the robot to respect children at a greater distance than adults. The STAC consists of two layers: the age-based layer, which represents personal space for different age groups, and the group-based layer, which represents group space.

#### 3.2.1. Age-Based Layer

**Figure 5:**
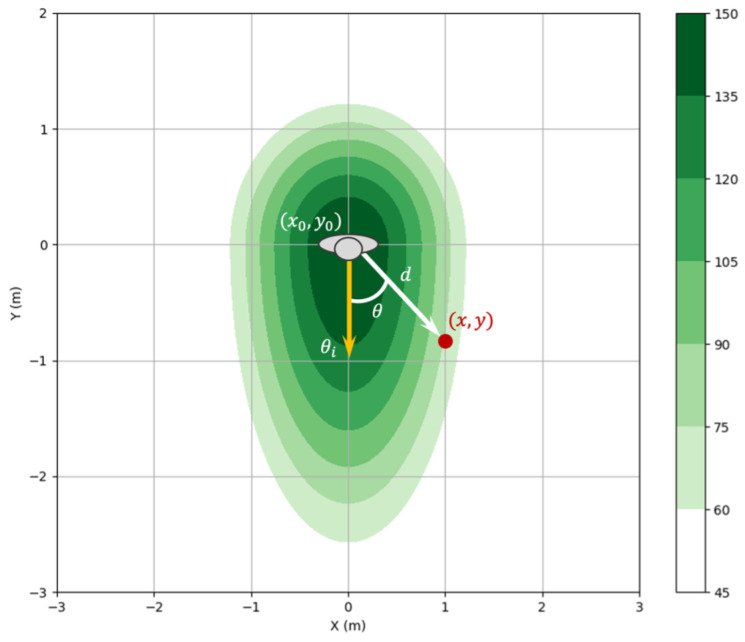
Example of the personal space around a human using an asymmetric Gaussian distribution.

We use an asymmetric Gaussian function to represent the personal space in mathematical form [11]. It is defined in Equation (2) and is visually illustrated in Figure 5. This modeling method helps the robot to move while ensuring an appropriate distance from the humans around it.

(2) $$\;f\left(x,y\right)=A\exp\left(-\left({\left(\frac{dcos(\theta -\theta_{i})}{\sqrt{2}\sigma_{x}}\right)}^{2}+{\left(\frac{dsin(\theta -\theta_{i})}{\sqrt{2}\sigma_{y}}\right)}^{2}\right)\right)$$

(3) $$\begin{array}{c} \;\sigma_{x}=\sigma_{i}\left(1+k_{v}v\right),\;\sigma_{y}=\sigma_{i} \end{array}$$

Using the human position from our Social Type Classification Pipeline, we define a danger zone centered on the human’s location $\left(x_{0},y_{0}\right)$. Given the moving direction of the person as $\theta_{i}=\mathrm{atan}2\left(v_{y},v_{x}\right)$, we calculate the cost value as the difference between the distance and direction of the person and any cell (x,y) on the costmap, where *d* is determined by $d=\sqrt{{\left(x-x_{0}\right)}^{2}+{\left(y-y_{0}\right)}^{2}}$ and θ is by $\theta =\mathrm{atan}2(\left(y-y_{0}\right),\left(x-x_{0}\right))$. The value decreases as it goes further from the person and adjusts based on the orientation difference between the person and the cell. The parameters of the Gaussian function, $A,\sigma_{x},\sigma_{y}$, represent the amplitude of the function and the standard deviation in the horizontal and vertical directions, respectively, which determine the size and shape of the personal space around the person. This zone of danger scales with the direction of the person’s movement, and, the faster the person moves, i.e., the greater the speed, the wider this zone of danger becomes. This is regulated through the scaling factor $k_{v}$, which adjusts the shape of the Gaussian function depending on the human’s speed and is shown in Equation (3). Through all this, the robot can accurately determine the personal space around people and move carefully between them accordingly. This is essential for the robot to respect social manners and personal space during interactions with people. Ref. [14] studied proxemics with a robot regarding participants’ age and found that children tended to stand further away from the robot than adults. This study suggests differences in proxemics between children and adults. Therefore, in this paper, we propose a new approach to make children’s personal space more extensive than that of adults. The size and shape of the personal space are based on the basic parameter $\sigma_{0}$, and Equation (4) describes how to use this parameter to differentiate the size of the personal space for children and adults. This takes into account the different proxemic needs of children and adults.

(4) $$\begin{array}{c} \;\sigma_{i}=\left\{\begin{array}{cc} \sigma_{0}, & \mathrm{if}\mathrm{adult}\\ 1.4\sigma_{0}, & \mathrm{if}\mathrm{child} \end{array}\right. \end{array}$$

The personal space parameter $\sigma_{0}$ for adults is set based on the proxemics. This theory takes the average distance an adult maintains in their general social interactions with strangers as a reference to their personal space. For example, if an adult’s average distance in a typical interaction with a stranger is 1.2 m, this distance is set to $\sigma_{0}$. On the other hand, research in Ref. [14] shows that children keep a distance of about 1.7 m on average when interacting with a robot. It is relatively longer than adults’ distance with a robot, so we should consider children’s personal space to be about 1.4 times larger than that of adults. Therefore, the personal space parameter for children is set to 1.4 times that of adults. This approach respects the difference in proxemics between children and adults and contributes to a more accurate interaction design.

**Figure 6:**
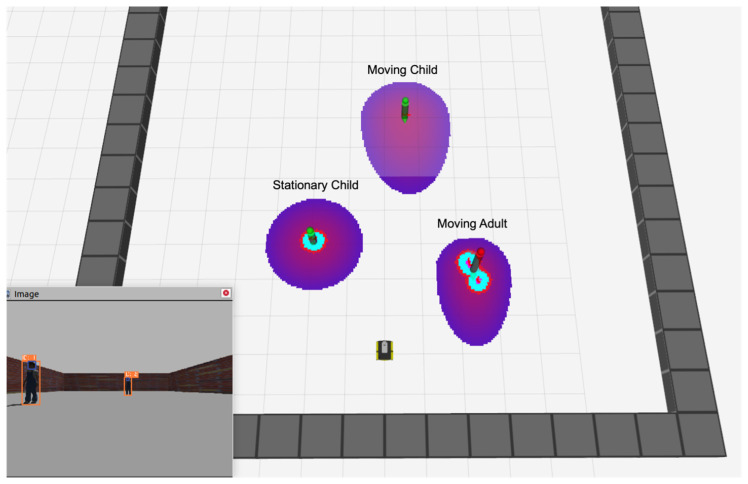
The result of applying an age-based layer. The personal spaces are shown at different sizes for a moving adult, a moving child, and a stationary child.

Figure 6 shows the result of applying the age-based layer. Notice the difference in the size of the Gaussian distributions applied to adults and children, particularly that the distribution for a moving child is extended in the moving direction. Using this information, the robot can adapt its path to avoid getting too close to the child.

#### 3.2.2. Group-Based Layer


(5) $$f_{G}\left(x,y\right)=A_{G}\exp\left(-\left({\left(\frac{dcos(\theta -\bar{\theta_{i}})}{\sqrt{2}\sigma_{x}^{G}}\right)}^{2}+{\left(\frac{dsin(\theta -\bar{\theta_{i}})}{\sqrt{2}\sigma_{y}^{G}}\right)}^{2}\right)\right)$$

(6) $$\begin{array}{c} \;\sigma_{x}^{G}=\sigma_{G}\left(1+k_{v}\bar{v}\right),\;\sigma_{y}^{G}=\sigma_{G} \end{array}$$

**Figure 7:**
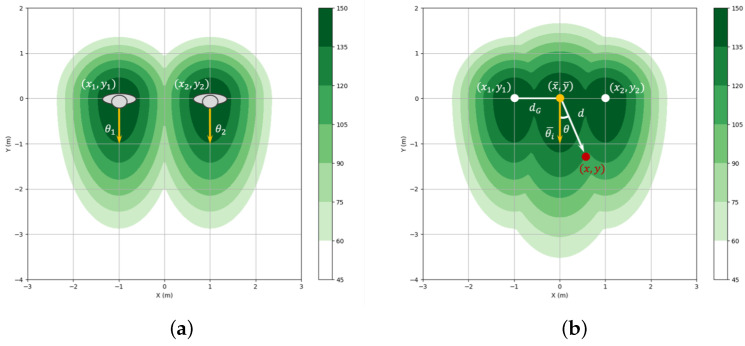
Asymmetric Gaussian distribution illustrating (**a**) two individuals’ personal spaces and (**b**) the integration of personal and group spaces.

To ensure human comfort in the context of human standing and moving in groups, the robot should also respect the group space shared by the group members. Compared to the age-based layer, represented based on the position and heading of individuals, shown in Figure 7a, the group-based layer captures the dynamics of the group of people shown in Figure 7b. It represents the group space based on the center position of the group $\left(\bar{x_{0}},\bar{y_{0}}\right)$ and the average direction $\bar{\theta_{i}}$ in which the group is moving together. Group space is defined similarly to individual space and is shown in Figure 7b and Equation (5). The group space is formed at the center of two individuals and, like the individual space described in Section 3.2.1, computes the cost values around the group members by comparing a random cell in the cost map with the group’s center position and moving direction. Here, *d* is determined by $d=\sqrt{{\left(x-\bar{x_{0}}\right)}^{2}+{\left(y-\bar{y_{0}}\right)}^{2}}$, and θ is by $\theta =\mathrm{atan}2(\left(y-\bar{y_{0}}\right),\left(x-\bar{x_{0}}\right))$. The parameter $\sigma_{G}$, which determines the size of the space, is defined by the distance $d_{G}$ from the group’s center to the farthest group member and the scaling factor $k_{G}$. That is, it is calculated with $\sigma_{G}=k_{G}d_{G}$. The wider the group members are separated, i.e., the larger *d* is, the larger $\sigma_{G}$ is, and the wider the danger zone around the group. It helps the robot keep a safe distance from all group members as it plans its path, considering the group.

**Figure 8:**
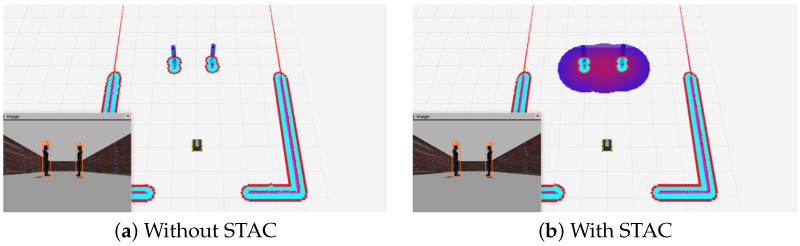
A comparison of the results with and without the STAC.

Figure 8 shows a comparison of the results with and without the STAC, simulating a situation where two people are talking in a virtual environment, Gazebo. Figure 8a shows without STAC, which does not capture the interaction between the two people, and the area between them is shown as free space for the robot to pass through, while Figure 8b is the result of with STAC, which detects both the individual and group space of the two people and shows the space where the robot cannot pass through, i.e., the lethal space. The proposed costmap layer is released as an open-source repository on GitHub [56].

## 4. Experiments

We demonstrated on both a simulator and an actual mobile robot that the proposed method enabled the robot to arrive at its destination in a dynamic environment where humans move around without causing discomfort. By experimenting with the robot’s interactions with different types of humans, including children, adults, and human groups, we verified that our method provides more comfort for humans than the traditional method. It presents a significant challenge to existing navigation strategies that consider humans only as single objects.

We used the Navfn [57] as the global planner and the Dynamic Window Approach (DWA) [58] as the local planner, and the navigation settings were kept consistent throughout the experiments. The main comparison is between the traditional costmap (obstacles and inflation layer) with and without the Social Type-Aware Costmap (STAC). Also, due to the child’s small size, the LiDAR cannot detect the child’s legs, so the camera holds the last detection position for 4 seconds in case the child is out of range. A video with our experimental results is available at https://youtu.be/LywBJ9KIxSE, accessed on 24 June 2024.

### 4.1. Robot Specifications

**Figure 9:**
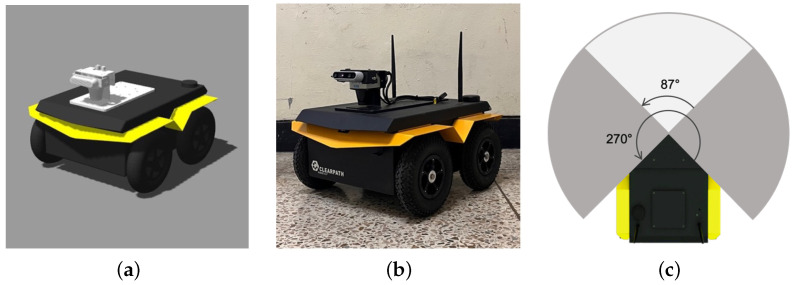
(**a**) The simulated robot model used in the experiment, (**b**) the real robot, and (**c**) the field of view (FoV) of the sensors mounted on the robot. The gray area depicts the range of the LiDAR sensor, and the white area depicts the camera sensor.

As shown in Figure 9a,b, the mobile robot used in both simulations and real-world experiments is Jackal [59], which is equipped with a 2D LiDAR and RGB-D sensor. The LiDAR sensor can measure objects between 0.05 m and 25 m and has a field of view of 270 degrees. We used Intel RealSense D435 as the RGB-D sensor, which has a minimum depth capability of 10.5 cm and supports depth/RGB frame rates up to 90/30 fps. The camera provides sensor resolutions up to 1280 × 720 for depth and 1920 × 1080 for RGB, with the field of view (FoV) being 87° × 58° × 94° for depth and 69° × 42° × 77° for RGB. Figure 9c shows a comparison of the field of view of the LiDAR and camera installed on the robot. The LiDAR sensor has a field of view of 270 degrees and the RGB-D camera has a field of view of 87 degrees for depth.

### 4.2. Experimental Setup and Scenarios

Our study includes simulation experiments to evaluate the framework with the Gazebo and the Robot Operating System (ROS) and real-world experiments to validate the performance of the proposed framework in social space. The simulation test environment was a corridor-like environment with child and adult models. To simulate the behavior of social agents, we used pedsimROS [60], an ROS package that models walking pedestrians using the Social Force Model [61]. They were set to recognize the robot and avoid it if necessary, and the child model was moved slower than the adult model.

We designed three test scenarios in which the robot had to move from the initial position to the goal while passing an adult, a child, and a human group. Each of these scenarios was conducted five times to ensure the consistency and reliability of the results. These scenarios were designed to evaluate how socially appropriate the robot interacts with humans with and without the Social Type-Aware Costmap (STAC). With this comparison, we can see how the use of the STAC affects the robot’s social type-aware navigation.

The real-world experiments consisted of 8 scenarios, each involving the robot interacting with an adult and a child and group interactions with two participants. The group interaction experiments included cases with two adults and cases where one participant was a child and the other an adult. Each scenario was designed for two different situations: when the participants were stationary and moving. The more detailed description of the scenario and its illustrations is presented in Section 4.4. It allowed us to observe how the robot reacted in different social situations. All experiments were compared between traditional navigation methods and using the STAC to evaluate how the robot navigated. The STAC parameter settings for simulation and real-world environments are shown in Table 1.

### 4.3. Simulation Experiment Results

**Figure 10:**
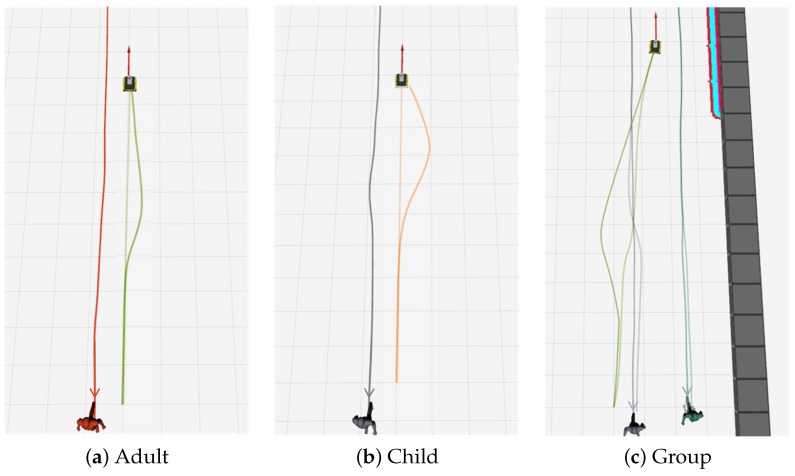
Trajectory results of the robot and humans in three simulated passing scenarios displayed in Rviz.

Figure 10 shows the trajectories of the robot and human model in the simulation experiment. Here, the robot’s destination is indicated by the red arrow. In Figure 10a, the red line shows the trajectory of the adult model and the green color shows the trajectory of the robot, where the light green is the trajectory of the robot when using the traditional costmap alone and the dark green is the trajectory when applying our proposed STAC method. Figure 10b shows the trajectory when the robot interacts with the child model, where the gray line shows the movement trajectory of the child model, and the light and dark orange show the robot trajectory when the traditional and STAC methods are applied, respectively. Figure 10c shows the trajectory of the robot avoiding an adult model moving in a group, comparing the results of the traditional and STAC methods. Here, the gray and cyan colors indicate the trajectories of the group members, the green indicates the trajectory of the robot, the light color indicates the traditional method, and the dark color indicates the trajectory when using the STAC method.

From these comparisons, we can see that, when using the STAC method, the robot moves with a clearer avoidance of the humans. In particular, when comparing Figure 10a,b, we can see that the robot tends to avoid the child model with a wider trajectory compared to the adult model. In addition, Figure 10c shows that the robot avoids the group more effectively when applying STAC than when using the traditional method, and, notably, we can see that the human nearest to the robot (light gray trajectory) tries to avoid the robot. The comparative analysis of these trajectories demonstrates the effectiveness of our approach on the robot’s avoidance strategy with respect to humans. More detailed numerical results can be found in Table 2. We used the following indices to evaluate and compare the methods.

- Navigation Time (NT): The traveling time until the robot arrives at the goal position [s];
- Path Length (PL): The total distance of the robot until it arrives at the goal position [m];
- Minimum Distance (MD): The minimum distance to human in all cases [m];
- Invasion Distance (ID): The total distance the robot moved while invading the personal space (1.2 m for an adult; 1.7 m for a child) [m].

Note that all the metrics except for minimum distance are average values.

**Table 2:** Simulation results.

Social Type	Method	NT [s]	PL [m]	MD [m]	ID [m]
Adult	Traditional	**22.39**	**9.51**	0.76	0.59
	STAC (Ours)	22.69	9.59	**1.11**	**0.15**
Child	Traditional	**22.23**	**9.51**	0.86	1.20
	STAC-A	22.77	9.65	1.23	0.99
	STAC (Ours)	23.62	9.75	**1.46**	**0.68**
Group	Traditional	31.69	**12.68**	0.41	**0.19**
	STAC (Ours)	**30.28**	12.91	**1.00**	0.33

#### 4.3.1. Adult

When using only the traditional costmap, the robot considered the adult as an obstacle and followed the shortest path to the goal, as shown by the light green line in Figure 10a. It tended to approach without any specific avoidance behavior that causes discomfort to the adult. However, MD and ID, indicators of human discomfort were found to be 0.76 m and 0.59 m, respectively, where MD means that the robot was closer to the person by 0.76 m. ID means that the robot invaded about 0.59 m, the minimum distance of 1.2 m that a human tries to maintain with a stranger, which means that, with the traditional costmap, the robot could get too close to the personal space of an adult and cause discomfort.

On the other hand, when applying our proposed STAC system, the robot approached the human at a minimum distance of 1.11 m, which is slightly closer than the human personal space threshold of 1.2 m. Moreover, the minimum distance the robot approached into the personal space was reduced by 0.44 m compared to the traditional method. Although STAC did not fully respect the human’s personal space, it still represents a significant improvement in ensuring that the human does not feel uncomfortable with the robot’s behavior compared to the traditional costmap method, as shown by the green line in Figure 10a. In addition, the navigation time and path length increased by 0.3 s and 0.08 m, respectively, compared to the conventional costmap results, but the difference was insignificant. These results show that STAC can be applied to navigate without significantly reducing navigation efficiency while respecting personal space and ensuring that humans are not uncomfortable with the robot’s behavior in human–robot interactions.

#### 4.3.2. Child

In the child–robot interaction scenario, we compared the traditional costmap, STAC, and STAC-A, which used the same settings as adults. The results show that the traditional costmap performed best regarding NT and PL. However, STAC performed the best in terms of MD and ID, which are indicators of ensuring a safe distance to a child. When the child’s personal space was set to 1.7 m, the ID value was 0.68 m despite using STAC. This is because the costmap generated around the child was defined as a Gaussian with a radius of about 1.7 m, but the center of the costmap was set to high risk, and the edges were set to relatively low risk. This is because, if the entire costmap was set to high risk, the robot would not be able to generate a path and would stop. While STAC-A also performed well regarding personal space consideration, the STAC method is more appropriate as it provides a larger personal space for children to protect them from unexpected situations during interaction with the robot. Furthermore, despite the NT and PL of 23.62 s and 9.75 m, respectively, increases of about 1.4 s and 0.24 m compared to the traditional method, the difference is insignificant throughout the navigation. The important thing is that the results show that our method reliably detects children and fully understands the extended personal space of children compared to the experiment with adults, applying different avoidance strategies than adults. This result demonstrates that our proposed Social Type-Aware Navigation Framework effectively distinguishes between children and adults and applies appropriate navigation strategies for each. As shown in Figure 10a,b, it is clear that the robot takes different trajectories when interacting with an adult and a child.

#### 4.3.3. Group

In a group interaction with a robot, we set the goal to the center of the group members. Using the traditional method, the robot planned the shortest path through the group’s center, which caused people to move to avoid the robot, as shown by the light green line in Figure 10c. While this approach may appear to reduce PL, increase NT, and improve ID values, this is actually because the robot had to stop while driving between people. The MD of 0.41 m may be a sign that the robot caused discomfort rather than providing comfort.

In contrast, when STAC is applied, the robot plans its path in a way that recognizes and respects personal and group space. As shown by the green line in Figure 10c, this allows people to move while maintaining their original trajectory, providing the robot a way of behaving that considers social etiquette. In particular, the traditional approach of trying to drive through the middle of a group is contrary to social etiquette, which is an important consideration for robot operation in public spaces. Using STAC, the robot can plan its avoidance path, reducing NT while achieving good performance in MD and ID, making human–robot interactions more natural and comfortable.

### 4.4. Real-World Experiments

#### 4.4.1. Individuals

**Figure 11:**
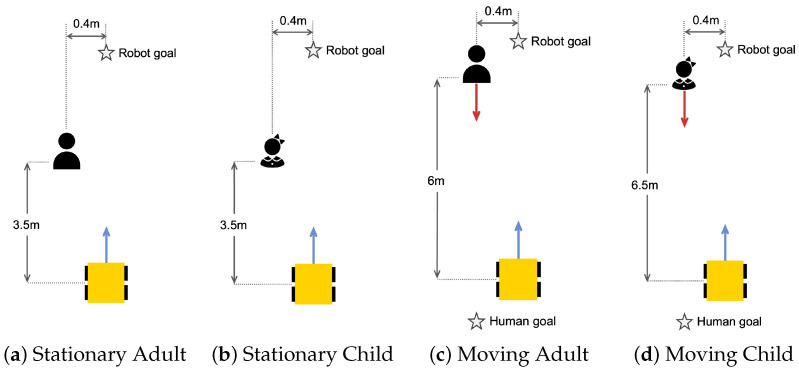
Real-world experiment scenarios for individual–robot interaction.

The real-world individual experimental scenarios were designed for two social types, an adult and a child, as illustrated in Figure 11. The experiments consisted of four different scenarios, including situations where the human is standing or moving, with a distance of about 0.4 m between the robot and the human. The robot navigates from a starting to a ’Robot goal’. The human standing is located 3.5 m vertically and 0.4 m horizontally away from the robot’s starting point, which can be seen in Figure 11a,b. On the other hand, in the scenario where the human is moving, the human and the robot start from the same point. The human moves about 6 m vertically and 0.4 m horizontally to the ’Human goal’, as shown in Figure 11c,d. By experimenting and comparing the robot’s response in these different situations using only the traditional costmap and the proposed STAC technique, we evaluated the performance of our framework in detecting the human and moving to the goal while respecting the personal space of different social types.

In the real-world experiments, we found that, across all scenarios, NT and PL, the navigation efficiency metrics as shown in Table 3 were best when using only the traditional costmap. However, STAC achieved better performance on MD and ID, indicators of human comfort. In the scenario where an adult is stationary, the traditional costmap kept a minimum distance of 0.66 m from the human following the planned path without any consideration for the human, and the total distance traveled within 1.2 m of the human’s personal space was measured to be 3.16 m. While the minimum distance of 0.66 m is shown numerically, this only takes into account the distance between the center of the robot and the human, which means that, in reality, given the width of the Jackal is 430 mm, the human may feel like the robot came within about 0.45 m. This is the general distance a human would keep from family or friends. In contrast, when STAC was applied, the robot considered the personal space when planning its path, ensuring that the minimum distance from the person was 1.09 m, and invaded the personal space while moving 2.80 m. A 1.2 m personal space around the person was not perfectly protected because giving a high-risk value to a space of this size would cause the robot to stop and be unable to generate a path.

**Figure 12:**
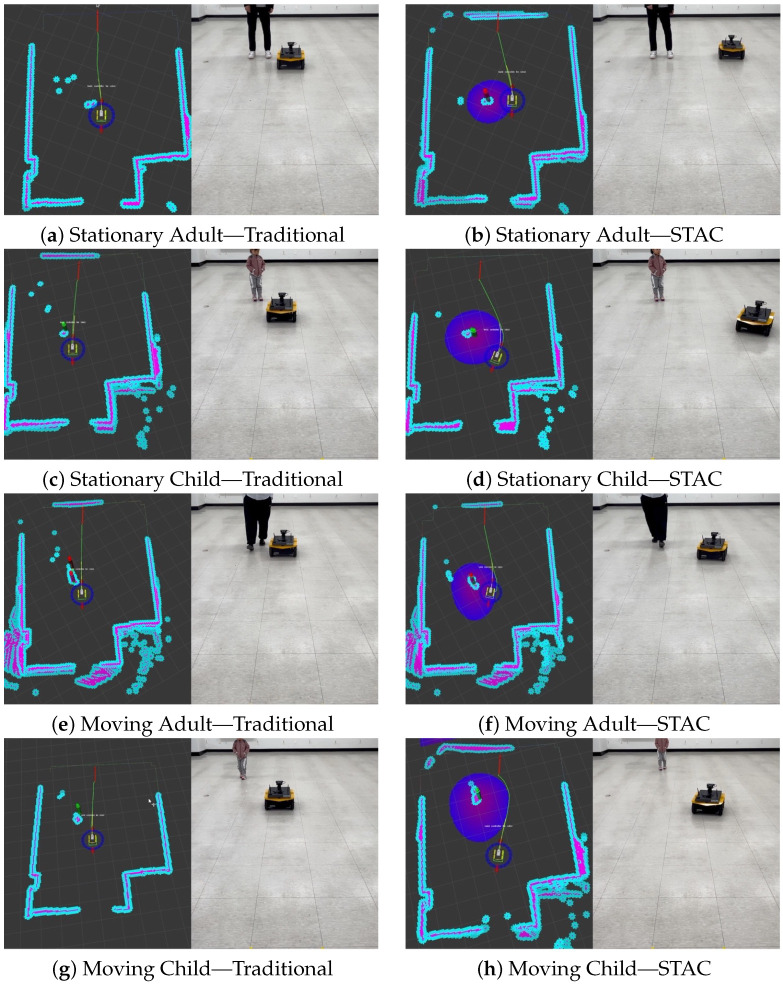
Experimental results in the real world with individuals. The left side of the figure is a snapshot of the Rviz visualization. The first row shows using the traditional costmap; the second row shows using STAC.

In the scenario where a child is standing, the traditional method approached the child with a minimum distance of 0.38 m, resulting from moving closer to the child than the adult due to the child’s smaller size. However, with STAC, the personal space applied around the child was set to be larger than that of the adult, allowing the robot to pass around the child more safely, resulting in a minimum distance between the robot and the child of 1.61 m. This means that a larger personal space was adopted even when compared to the experiment with the adult. When the child’s personal space was set to 1.7 m, the robot invaded this space while traveling 3.12 m with the traditional method, but STAC reduced this to 0.34 m. These results can be seen in Figure 12b,d, where the robot follows a safer trajectory around the child. It is important to note that planning a safe trajectory prioritizes safety even at the expense of some navigation efficiency, as shown by the increase in NT of about 3.35 s and PL of 0.16 m compared to the traditional method. This is a small tradeoff for safety. For the moving adult scenario, the traditional method resulted in driving at 0.41 m, closer than the stationary adult scenario, which caused the human to take action to avoid the robot. This approach did not respect the personal space, making the human feel uncomfortable. However, with STAC, the robot recognized the increased personal space in the direction of the human’s movement and moved away from it, maintaining a distance of 0.88 m from the adult. The experiment with the child showed similar results, maintaining a distance of at least 1.47 m from the child, which is significantly greater than the 0.70 m measured with the traditional method.

Overall, in the scenario of interacting with a single person, traditional methods could not maintain the required minimum distance due to their approach of considering people as obstacles and following the shortest path without taking any special avoidance measures. This approach was particularly challenging in environments with vulnerable individuals, such as children. Because of their small size, traditional methods only recognize children as small obstacles and tend to navigate at closer distances than adults. In contrast, STAC respects personal space, especially in interactions with children. We use cameras to identify children and apply appropriately sized costmap to them, even if they are too small to be detected by LiDAR sensors. Once a child is detected, the costmap is kept at the child’s last known location for a short time, even if the camera no longer detects the child, to ensure a safe distance between the child and the robot. Our approach allows the robot to respect better personal space in interactions with all people, not just children, and enables safe and comfortable navigation.

#### 4.4.2. Human Group

**Figure 13:**
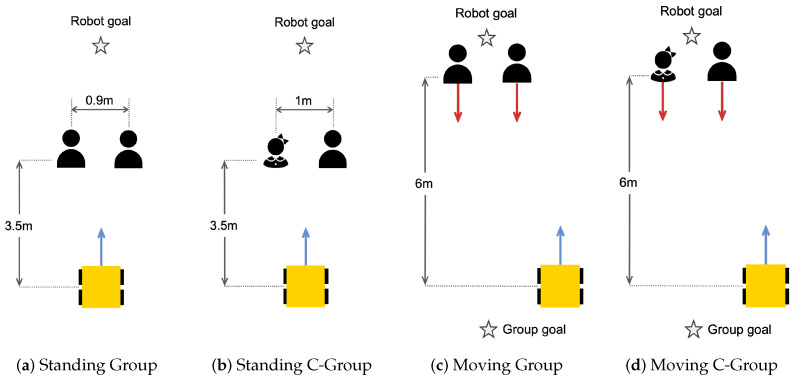
Real-world experiment scenarios for group–robot interaction.

The real group experiment scenario is shown in Figure 13; the experiments focused on setting the distance between the robot and the group members, with a distance of approximately 1 m between group members and either 3.5 or 6 m from the robot. The experiments were tested for two cases: a group consisting entirely of adults and a group including one child. The robot moves from a given starting position to the ’Robot goal’. When the group is standing and interacting with each other, the robot starts 3.5 m away from the group, and the robot goal is set at a position across the center of the group, as shown in Figure 13a,b. In the group moving scenario, the group members start about 6 m away from the robot and move to the ’Group goal’, where the robot goal is also located across the group’s center. It can be seen in Figure 13c,d. These experiments are designed to compare the robot’s behavior when only applying the traditional costmap and STAC methods. This allows us to evaluate how the robot reacts differently to a group consisting of only adults versus a group containing children and how efficiently the robot can access the goal set by crossing the group’s center.

**Figure 14:**
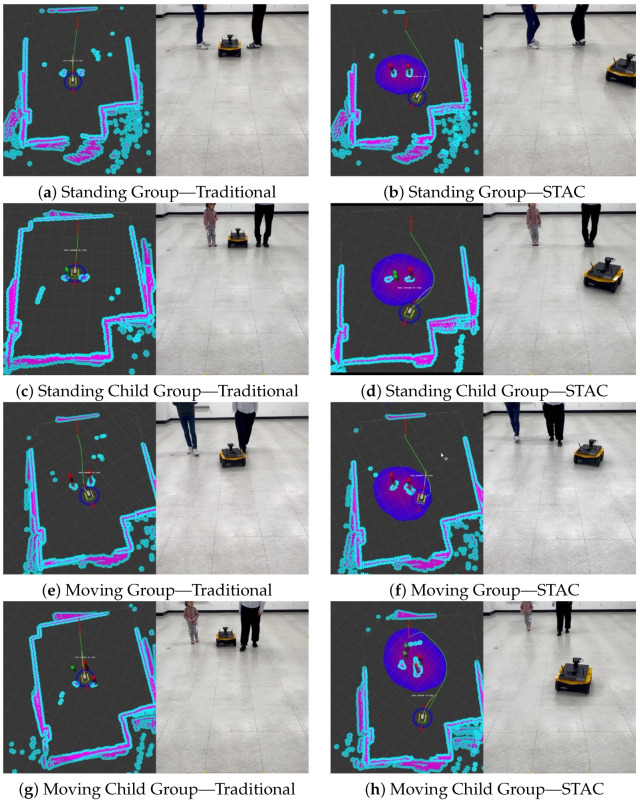
Experimental results in real-world group interaction. The left side of the figure is a snapshot of the Rviz visualization. The first row shows using the traditional costmap; the second row shows using STAC.

The behavior of the robot and its global path are shown in Figure 14 for all scenarios in the group interaction experiments. The robot using the traditional costmap was utterly unconscious of group interaction and chose paths that crossed between group members, whether they were stationary or moving. This passing behavior between group members is particularly problematic in scenarios involving children. Passing by children is problematic because they can be more sensitive and unpredictable and require more space to ensure their safety and comfort. When a robot invades the personal space of a group, including children, it can lead to discomfort and safety issues. With STAC, on the other hand, the robot can regard both the personal space of the group members and the group space, which is the space between the group members. This allows the robot to choose a path that avoids disrupting the group’s interaction and moves without moving through the group.

The results of the experiments are detailed in Table 4. In the experiment with a group including a child, the Invasion Distance value was measured by adjusting the personal space required by the child from 1.7 m to 1.2 m, as opposed to the previous experiment. With a group of stationary adults, using the traditional method, the robot traveled at a minimum distance of 0.61 m from the person. However, with STAC, the robot traveled at a minimum distance of 1.10 m. This distance makes people more comfortable by moving through a path that largely avoids the group. Although STAC did not fully respect the personal space of 1.2 m for the group, choosing a path that avoided the group instead of driving through resulted in a safer and more comfortable interaction. In the scenario with a group including a child, there may not be much difference between the traditional method and STAC in the minimum distance (MD), measuring 0.44 and 0.62 m, respectively. This is because group space is adapted between group members, and each individual is given their own space. However, the significant difference is that STAC travels around the group space rather than having the robot pass directly between people. This approach makes the child and group members feel more comfortable with the navigation than having the robot pass directly between group members, especially for a group with a child.

In the moving adult group experiment, when using the traditional costmap, the robot chose a path that went through the group, avoiding people who were closer to the robot. The measured MD and ID in this scenario were 0.51 m and 1.14 m, respectively. However, when applying our proposed STAC, the robot was proactively aware of this and modified its path to avoid it, with a costmap that considered personal and group space. The MD was found to be 0.63 m, although it did not respect the human’s personal space of 1.2 m. This is a significant improvement as it allows the human to move without having to modify his/her path, considering that it is not always possible to ensure absolute personal space in a dynamic environment. When a child and an adult were moving together, the robot, using only the traditional costmap, traveled between them and maintained a minimum distance of 0.33 m from the person. However, the MD increased to 0.86 m for the robot using STAC. These results show that STAC is more respectful of personal space than traditional methods and is particularly effective at ensuring a safe distance between robots and people in groups containing a child, meaning that the robot will follow a safer trajectory around the child. Thus, STAC shows more respect for human interaction and works to provide driving trajectories that ensure the safety and comfort of all group members.

## 5. Conclusions

In this paper, we propose a new framework, the ’Social Type-Aware Navigation Framework’, for robots to classify individuals and groups within a social space and navigate while respecting the appropriate social distance. This framework utilizes sensors commonly mounted on robots, such as a camera and LiDAR, to detect and track people in real time, classify them by social type, and apply social distances for each type to make people feel comfortable and safe with the robot’s driving. The ‘Social Type-Aware Costmap’ proposed in this research is developed as a compatible plugin to the ROS-based navigation stack, which can be easily integrated into various robot systems and path-planning algorithms. The robot can recognize the behavior patterns based on age and group and adopt suitable avoidance strategies for each situation to ensure the safety of the interaction. This is expected to increase the social acceptance of robots, providing a new perspective in the field of human–robot interaction, with a particular focus on interactions with children. By recognizing vulnerable subjects such as children and maintaining an appropriate social distance for them, this research provides a basis for a robot to be perceived as a social being. Future research will include the consideration of not only children but also other vulnerable individuals such as the elderly and wheelchair users. Additionally, it is important to consider the size of the robot as a significant variable in developing the costmap and planning avoidance strategies. While our current study focused on a relatively small robot, future research should explore how different robot sizes affect human–robot interactions and the effectiveness of the proposed navigation framework. The goal is to further enhance the social acceptability of robots by enabling them to recognize and act on a wider range of people. However, the definition of personal space can vary depending on cultural and individual differences, so this is an area for continuous research and development in human–robot interaction. This is a significant challenge in enhancing the ability of robots to understand and adapt to social and cultural contexts.

## Figures and Tables

**Table 1:** Parameter settings.

Parameter	Simulation	Real World
*A*	150	125
$\sigma_{0}$	0.8	0.5
$\sigma_{G}$	0.7	1.0
$k_{v}$	5	5
$A_{G}$	170	125
$k_{G}$	1.5	1.3

**Table 3:** Individual experiment results.

Scenario	Method	NT [s]	PL [m]	MD [m]	ID [m]
Stationary Adult	Traditional	**18.79**	**6.92**	0.66	3.16
	STAC (Ours)	21.06	7.09	**1.09**	**2.80**
Stationary Child	Traditional	**19.85**	**6.56**	0.38	3.12
	STAC (Ours)	23.20	6.72	**1.61**	**0.34**
Moving Adult	Traditional	**17.89**	**7.34**	0.41	1.31
	STAC (Ours)	21.57	7.70	**0.88**	**0.83**
Moving Child	Traditional	**20.02**	**6.73**	0.70	3.22
	STAC (Ours)	21.40	7.30	**1.47**	**0.62**

**Table 4:** Human group experiment results.

Scenario	Method	NT [s]	PL [m]	MD [m]	ID [m]
Standing Group	Traditional	**17.75**	**6.71**	0.61	2.19
	STAC (Ours)	21.27	7.36	**1.10**	**0.58**
Standing C-Group	Traditional	**18.20**	**7.30**	0.44	4.30
	STAC (Ours)	22.75	7.68	**0.62**	**3.23**
Moving Group	Traditional	**20.16**	**7.05**	0.51	1.14
	STAC (Ours)	21.15	7.59	**0.63**	**0.97**
Moving C-Group	Traditional	**20.32**	**7.01**	0.33	2.68
	STAC (Ours)	23.75	7.94	**0.86**	**0.85**

## Data Availability

Data are contained within the article.
